# Advances in Imaging of Plant Ca^2+^ Signaling

**DOI:** 10.3390/biom16081193

**Published:** 2026-08-15

**Authors:** Zhenzhong Tang, Shuangyuan Fan, Guanhong Lin, Tangtao Yuan, Shuang Yang

**Affiliations:** College of Life Sciences, Northeast Forestry University, Harbin 150069, China

**Keywords:** plant calcium signaling, genetically encoded calcium indicators, calcium transport systems, abiotic stress, plant immunity

## Abstract

Calcium ions (Ca^2+^) function as ubiquitous second messengers that translate environmental and developmental cues into spatially and temporally defined cellular responses in plants. This review summarizes the cellular architecture and molecular mechanisms that generate, shape, and terminate Ca^2+^ signals, with emphasis on plasma-membrane channels, intracellular stores, pumps, exchangers, and organelle-associated transport systems. We also examine the development of live Ca^2+^ indicators, from chemical dyes and aequorin to ratiometric and single-fluorophore genetically encoded calcium indicators, and discuss principles for selecting sensors for different tissues and subcellular compartments. Recent studies have applied these tools to abiotic stress, plant immunity, polar growth, development, symbiosis, and systemic signaling. Accurate quantitative imaging nevertheless requires careful matching of sensor properties to the target cellular environment and rigorous control of motion, spectral interference, and analytical procedures. Combining improved indicators with advanced microscopy, genetic validation, and standardized data analysis should help connect distinct Ca^2+^ signatures with their molecular origins and physiological roles.

## 1. Introduction

Throughout their life cycle, plants are exposed to temperature fluctuations, salinity, mechanical perturbation, and pathogen attack. The cytosolic Ca^2+^ concentration ([Ca^2+^]_i_) changes rapidly after stimulus perception, often representing one of the earliest events in signal transduction and contributing to subsequent defense responses, developmental regulation, and environmental acclimation [1,2,3]. Compared with responses that depend on hormone accumulation or transcriptional regulation, changes in [Ca^2+^]_i_ occur much earlier and therefore provide an important entry point for dissecting early signaling events in plants.

The formation of plant [Ca^2+^]_i_ signals depends on steep transmembrane concentration gradients. Under resting conditions, [Ca^2+^]_i_ is maintained at a low level, whereas the apoplast, vacuole, endoplasmic reticulum, and other subcellular compartments serve as major Ca^2+^ stores [2,4]. After stimulation, Ca^2+^ channels, pumps, and exchangers located on different membrane systems alter [Ca^2+^]_i_ and generate spatially and temporally distinct Ca^2+^ patterns. Stimulus-induced changes in [Ca^2+^]_i_ are therefore not simply increases in bulk cytosolic concentration; rather, they comprise specific combinations of amplitude, frequency, duration, and spatial distribution. These patterned dynamics are commonly referred to as “Ca^2+^ signatures” [3,5]. Distinct stimuli generate different Ca^2+^ signatures, which may enable plant cells to discriminate environmental information and mount stimulus-specific responses.

Ca^2+^ signaling involves not only signal generation but also the perception, recognition, and decoding of the signal. Plants contain multiple classes of Ca^2+^-binding proteins and sensors, including calmodulin (CaM), calmodulin-like proteins (CMLs), calcineurin B-like proteins (CBLs), and calcium-dependent protein kinases (CDPKs/CPKs) [6,7,8]. Ca^2+^ binding induces conformational changes in these proteins, which in turn regulate ion transport, protein phosphorylation, cellular metabolism, and gene expression. In this way, transient Ca^2+^ signals are converted into longer-lasting cellular responses.

Plant Ca^2+^ signaling encompasses signal generation, membrane-system regulation, downstream decoding, and the restoration of homeostasis, thereby contributing broadly to plant physiology. Abiotic stresses, including salinity, low temperature, drought, heat, and mechanical stimulation, can rapidly elevate [Ca^2+^]i, alter reactive oxygen species metabolism and osmotic adjustment, and induce stress-responsive gene expression [3,9,10]. In plant immunity, Ca^2+^ influx is an early signaling event. Following recognition of pathogen-associated molecules, extracellular Ca^2+^ rapidly enters the cytosol and is coupled to the oxidative burst, mitogen-activated protein kinase (MAPK) activation, and defense-gene transcription, thereby contributing to both pattern-triggered and effector-triggered immunity [11,12]. Beyond stress and immunity, Ca^2+^ signals regulate pollen-tube elongation, guard-cell movement, root-hair growth, symbiotic interactions, and organ development, underscoring their broad roles in plant growth, development, and environmental adaptation [13,14].

Despite major advances in plant Ca^2+^ signaling research, the precise dynamics of these signals remain difficult to resolve because of technical limitations. Plant cells possess thick cell walls, large vacuoles, and substantial autofluorescence, all of which interfere with live-cell Ca^2+^ imaging and complicate quantitative analysis at high spatial and temporal resolution. Ca^2+^ signals also develop rapidly and are highly sensitive to mechanical disturbance; even minor perturbations introduced during sample preparation or imaging can distort the native response. In addition, pronounced differences in the Ca^2+^ environment among cell types and subcellular compartments generate strong spatial specificity even under the same stimulus. Consequently, conventional bulk biochemical measurements cannot faithfully reconstruct the true spatiotemporal characteristics of Ca^2+^ dynamics [4,13].

These limitations have driven a shift from bulk measurements toward genetically encoded indicators and live-imaging platforms. Current research increasingly asks where Ca^2+^ signals originate, how long they persist, and how they move between cells, tissues, and subcellular compartments. Addressing these questions requires transport biology, sensor design, controlled stimulus delivery, and quantitative imaging to be considered together.

## 2. Cellular Architecture and Molecular Basis of Ca^2+^ Transport in Plant Cells

**Figure 1:**
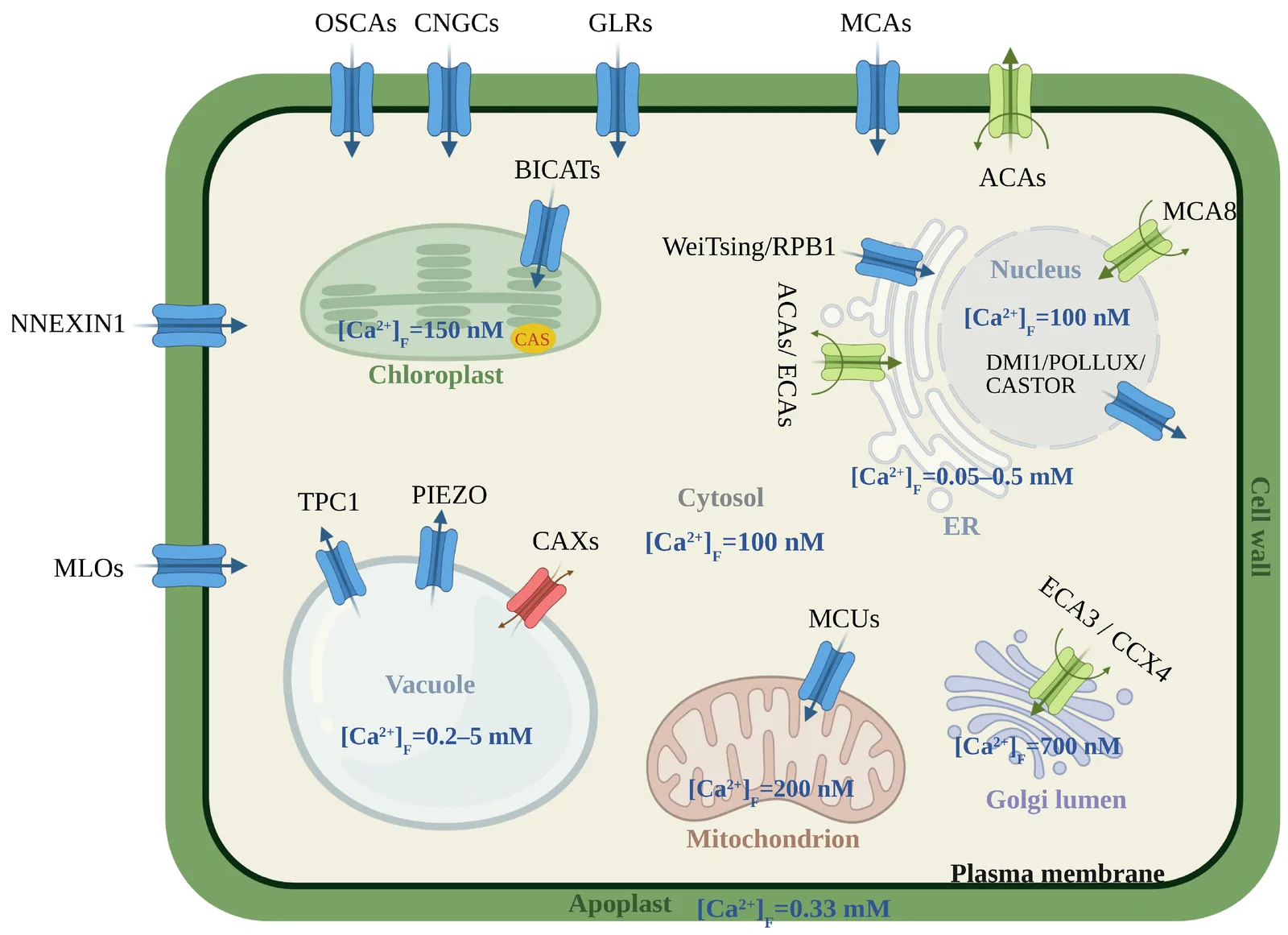
Subcellular distribution and classification of major Ca2+ channels and transport systems involved in plant Ca2+ signaling. Plasma membrane-localized channels mediate Ca2+ influx, whereas organelle-associated channels and transporters contribute to intracellular Ca2+ storage, release, and homeostasis. Representative free Ca2+ concentrations in different subcellular compartments are indicated. Abbreviations are defined in the main text. Created in BioRender. zz, T. (2026) https://BioRender.com/tj3qrt1. Arrows indicate the direction of Ca^2+^ transport across the respective membranes. Different colors are used to visually distinguish the transport proteins and do not represent Ca^2+^ concentration or transport magnitude.

The generation of Ca^2+^ signals in plant cells relies on steep concentration gradients across the plasma membrane and among subcellular compartments. Under resting conditions, free cytosolic Ca^2+^ is maintained at approximately 100 nM, whereas other compartments establish distinct Ca^2+^ microenvironments spanning submicromolar to millimolar ranges. Representative values include approximately 100 nM in the nucleus, 150 nM in the chloroplast stroma, 200 nM in the mitochondrial matrix, 700 nM in the Golgi lumen, 0.05–0.5 mM in the endoplasmic reticulum, 0.2–5 mM in the vacuole, and 0.33 mM in the apoplast (Figure 1) [2,3,4]. When plants perceive external stimuli, channels, pumps, and exchangers in different membrane systems are activated, resulting in extracellular Ca^2+^ influx, release from intracellular stores, or rapid Ca^2+^ resequestration and thereby altering cytosolic Ca^2+^ levels.

The plasma membrane and endomembrane systems perform distinct but complementary functions in Ca^2+^ signaling. Plasma-membrane transport is primarily associated with early perception of external stimuli, whereas endomembrane systems mediate Ca^2+^ release, sequestration, and redistribution among compartments. These processes determine signal duration, propagation range, and subcellular localization [13,14]. Thus, Ca^2+^ dynamics in plant cells arise from the coordinated activity of multiple compartments rather than from a single membrane system. Differences among membrane systems in localization, activation mechanism, transport properties, and basal free Ca^2+^ concentration contribute to the generation of diverse Ca^2+^ signatures. The principal transport systems and representative compartmental Ca^2+^ concentrations are summarized in Figure 1.

### 2.1. Plasma-Membrane Ca^2+^ Channels and Early Stress Perception

The plasma membrane is the primary cellular interface with the external environment, and many early Ca^2+^ signals originate at this site. Osmotic changes, mechanical stimulation, temperature stress, pathogen recognition, and tissue damage commonly alter plasma-membrane permeability. Conversion of an external stimulus into an intracellular Ca^2+^ signal therefore begins with ion transport across the plasma membrane. Among the best-studied plant plasma-membrane Ca^2+^ channels are cyclic nucleotide-gated channels (CNGCs). Several CNGC family members contribute to pathogen-induced Ca^2+^ influx and innate immune responses [15,16]. Because the CNGC family is large, individual members differ in tissue distribution and stimulus responsiveness, and functional redundancy among family members remains incompletely resolved.

Glutamate receptor-like channels (GLRs) are key plasma-membrane components in plant wound responses and systemic signal propagation. Following local tissue damage, glutamate released at the wound site activates GLR proteins in vascular-associated cells, triggering Ca^2+^ signals that spread through the tissue as long-distance Ca^2+^ waves. These signals contribute to the establishment of defense competence in distal tissues. Thus, plasma-membrane Ca^2+^ channels regulate not only ion transport at the single-cell level but also rapid signaling at the tissue scale [17].

For physical stimuli, members of the OSCA and MCA (Mid1-complementing activity) families are important components of osmotic and mechanosensory pathways [14]. However, the channel components that respond to different types of mechanical stimulation and the underlying molecular mechanisms remain only partially defined.

The biological significance of a Ca^2+^ signal depends not only on its activation but also on its timely termination. The plasma membrane therefore has a dual function: it mediates Ca^2+^ entry into the cytosol and, through autoinhibited Ca^2+^-ATPases (ACAs), exports Ca^2+^ to restore the resting cytosolic concentration. From signal initiation and attenuation to the re-establishment of ion homeostasis, the plasma membrane remains a central regulatory site.

ANNEXIN1 and MLO family proteins also contribute to plasma-membrane Ca^2+^ permeability. ANNEXIN1 mediates Ca^2+^ influx induced by reactive oxygen species and salt stress and participates in stress signaling [18,19]. MLO1, MLO5, MLO9, and MLO15 have been shown to possess Ca^2+^ channel activity and to contribute to the formation of the pollen-tube Ca^2+^ gradient and maintenance of pollen-tube integrity [20].

### 2.2. Dynamic Regulation and Transport Networks of Intracellular Ca^2+^ Stores

Plasma-membrane Ca^2+^ influx alone cannot explain the compartmentalized regulation of plant Ca^2+^ signals. The vacuole is one of the largest intracellular Ca^2+^ stores and plays a major role in shaping cytosolic Ca^2+^ dynamics. Two-pore channel 1 (TPC1) in the tonoplast is associated with ion release and stress-induced electrical activity and may contribute to vacuole-related Ca^2+^ dynamics [21]. Plant PIEZO homologs localize to the tonoplast and influence cytosolic Ca^2+^ oscillations, vacuolar morphology, and tip growth, although whether they directly mediate vacuolar Ca^2+^ release remains to be fully established [22].

In addition to releasing Ca^2+^, the vacuole participates in Ca^2+^ sequestration. Tonoplast Ca^2+^/H^+^ exchangers (CAXs) transport cytosolic Ca^2+^ back into the vacuole, thereby replenishing vacuolar Ca^2+^ stores and restoring cytosolic Ca^2+^ concentrations [23,24]. These exchangers directly influence the rate of signal attenuation and are essential components of intracellular Ca^2+^ homeostasis. Recent work showed that vacuolar CAX activity is controlled by two convergent phosphorylation pathways: CBL–CIPK signaling activated by elevated extracellular Ca^2+^ and FLS2–BAK1–BIK1/PBL1 signaling activated during immune perception both target an autoinhibitory serine cluster in CAX proteins, thereby coordinating cytosolic Ca^2+^ homeostasis with plant growth and immunity [25].

The endoplasmic reticulum is another important Ca^2+^ store in plant cells. Beyond serving as a Ca^2+^ reservoir, the ER luminal Ca^2+^ pool also supports protein folding and quality control. The Ca^2+^-binding molecular chaperone calreticulin contributes to ER Ca^2+^ buffering and protein-folding capacity, thereby linking ER Ca^2+^ homeostasis to secretory-pathway function [26,27]. The endoplasmic-reticulum-localized pentameric channel WeiTsing/RPB1 (Resistance to Plasmodiophora brassicae 1) is a plant-specific Ca^2+^-permeable cation channel that provides molecular evidence for an endoplasmic-reticulum-localized Ca^2+^ release pathway in plants [28]. Conversely, endoplasmic-reticulum-type Ca^2+^-ATPases (ECAs) and a subset of ACAs located on endomembranes pump cytosolic Ca^2+^ into the endoplasmic reticulum and Golgi apparatus, thereby maintaining local stores and influencing secretion and stress acclimation [3,14]. Within the secretory pathway, ECA3 has been proposed to mediate Ca^2+^/Mn^2+^ transport into the Golgi or related post-Golgi compartments, whereas CCX4 has been demonstrated to localize to the Golgi apparatus and contribute to Ca^2+^ homeostasis and osmotolerance [29,30]. Thus, the vacuole, endoplasmic reticulum, and Golgi do not merely store Ca^2+^; they actively mediate its release, sequestration, and redistribution.

### 2.3. Generation of Intracellular Ca^2+^ Signals and Re-Establishment of Ca^2+^ Homeostasis

Advances in compartment-targeted indicators and live imaging have revealed important roles for chloroplasts, mitochondria, and the nuclear envelope in plant Ca^2+^ regulation [31,32]. In contrast to early studies focused primarily on cytosolic Ca^2+^, accumulating evidence indicates that distinct organelles shape local Ca^2+^ microenvironments and couple Ca^2+^ dynamics to metabolic regulation and signal transduction. Chloroplast Ca^2+^ dynamics are associated with light responses, stress acclimation, and maintenance of chloroplast homeostasis. Chloroplast-localized bivalent cation transporters (BICATs), including BICAT1 and BICAT2, participate in chloroplast Ca^2+^ transport and influence stromal Ca^2+^ signaling [33]. The calcium-sensing receptor CAS also contributes to chloroplast-associated signaling [34,35]. In addition, KEA (K^+^ efflux antiporter) family proteins regulate K^+^/H^+^ exchange and stromal pH, thereby maintaining chloroplast ion homeostasis and creating an appropriate ionic environment for Ca^2+^ dynamics and related signaling processes [36]. However, the mechanistic relationships among these components, photosynthetic electron transport, reactive oxygen species accumulation, and stress responses remain incompletely understood.

Mitochondrial calcium uniporters (MCUs) transport Ca^2+^ into the mitochondrial matrix and contribute to redox balance and energy metabolism. Mitochondria also provide local Ca^2+^ buffering and participate in signal reshaping. Nevertheless, the composition and regulation of the plant mitochondrial Ca^2+^ transport network remain to be fully elucidated [37].

Nuclear-envelope-associated Ca^2+^ dynamics are particularly important in symbiotic signaling. The nuclear-envelope cation channels DMI1 (Does not Make Infections 1), POLLUX, and CASTOR are closely associated with the generation of perinuclear Ca^2+^ oscillations, which are a key signal during symbiotic recognition in legumes [32,38]. Nuclear-envelope Ca^2+^ pumps also contribute to this process. For example, the nuclear-envelope- and endoplasmic-reticulum-localized Ca^2+^-ATPase MCA8 promotes Ca^2+^ resequestration and is required to sustain repetitive nuclear Ca^2+^ oscillations [39].

The function of a Ca^2+^ signal depends not only on its induction but also on its subsequent attenuation. Clearance of cytosolic Ca^2+^ should not be viewed merely as restoration of homeostasis, because the rate of decline, signal duration, and the capacity of different compartments to sequester Ca^2+^ all contribute to the final dynamic pattern [13].

ATP-driven pumps on different membrane systems play central roles in restoring Ca^2+^ homeostasis. ACAs and ECAs transport Ca^2+^ from the cytosol to the apoplast or intracellular storage compartments, whereas gradient-driven CAX and CCX exchangers mediate Ca^2+^ sequestration and re-compartmentalization. Together, these systems return cytosolic Ca^2+^ to resting levels after stimulation and preserve the capacity for subsequent responses. Ca^2+^ pumps and exchangers are therefore not merely signal-termination components; they also help shape Ca^2+^ signatures.

## 3. Development of Ca^2+^ Indicators for Live Imaging in Plants

Building on advances in Ca^2+^ transport and compartmentalization, a central technical challenge has been to record rapid, compartment-specific Ca^2+^ dynamics in living plants. Compared with animal cells, plant tissues impose additional constraints on Ca^2+^ imaging. The cell wall can hinder the loading of exogenous indicators into intact tissues [2,4]. In addition, chloroplasts and cell-wall-associated components can generate substantial autofluorescence, increasing background signals and reducing fluorescence contrast [2,4]. Mechanical perturbations introduced during sample preparation, immobilization, or microscopy can themselves trigger Ca^2+^ responses and thereby confound interpretation of the native signal. Differences in basal free Ca^2+^ concentration among subcellular compartments further constrain detection accuracy. The concentration range spans several orders of magnitude, from approximately 100 nM in the cytosol and nucleus to millimolar levels in the vacuole and endoplasmic reticulum (Figure 1). Indicators therefore differ in affinity, response range, and stability across compartments, and a sensor optimized for the cytosol may saturate in a Ca^2+^-rich store. Consequently, optimization of plant Ca^2+^ imaging tools has shifted from simply increasing signal intensity or sensitivity toward an integrated emphasis on quantitative performance, tissue compatibility, subcellular targeting, and stability during long-term imaging.

**Figure 2:**
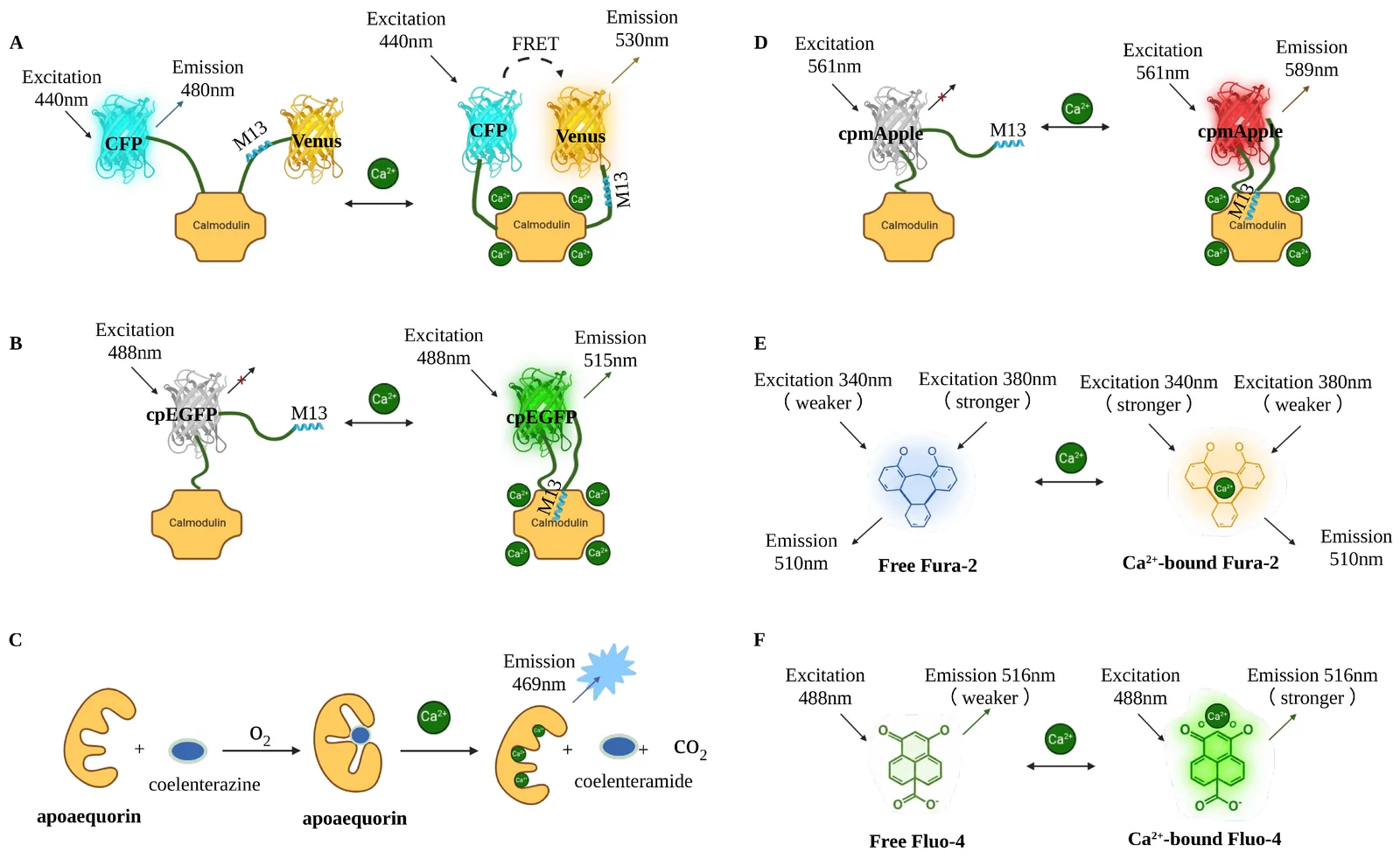
Molecular architectures and signal-generation mechanisms of representative Ca^2+^ indicators. Note: (**A**) Cameleon: Ca^2+^-dependent calmodulin–M13 association increases FRET from CFP to Venus. (**B**) GCaMP: Ca^2+^ binding stabilizes the M13–cpEGFP–calmodulin complex and increases green fluorescence. (**C**) Aequorin: Ca^2+^-dependent oxidation of coelenterazine produces blue luminescence near 469 nm. (**D**) R-GECO-type indicators generate Ca^2+^-dependent red fluorescence through cpmApple. (**E**) Fura-2 is a dual-excitation ratiometric dye: Ca^2+^ binding increases excitation at 340 nm and decreases excitation at 380 nm, with emission near 510 nm. (**F**) Fluo-4 is an intensity-based dye whose fluorescence near 516 nm increases after Ca^2+^ binding under 488-nm excitation. Abbreviations: CFP, cyan fluorescent protein; cpEGFP, circularly permuted enhanced green fluorescent protein; cpmApple, circularly permuted mApple; FRET, fluorescence resonance energy transfer. Created in BioRender. zz, T. (2026). https://BioRender.com/4v1gxhp. Solid arrows indicate excitation, emission, or reaction steps; the dashed arrow in panel (**A**) denotes FRET from CFP to Venus, and double-headed arrows indicate reversible Ca^2+^-dependent transitions.

Historically, plant Ca^2+^ imaging has progressed through three major classes of indicators: chemical fluorescent dyes, bioluminescent proteins, and genetically encoded calcium indicators (GECIs). These indicator types use distinct photophysical mechanisms and differ in signal intensity, temporal resolution, background interference, compartmental targeting, and suitability for continuous observation (Figure 2); they are therefore best suited to different biological questions [9,40,41,42].

### 3.1. Chemical Fluorescent Dyes and Aequorin-Based Ca^2+^ Imaging

Early plant Ca^2+^ imaging relied primarily on chemical fluorescent dyes. Fura-2 is a dual-excitation ratiometric indicator: Ca^2+^ binding shifts the excitation response from 380 toward 340 nm while emission remains near 510 nm, enabling quantitative ratio-based measurements (Figure 2E). Fluo-4 is a single-excitation, intensity-based indicator whose fluorescence near 516 nm increases markedly after Ca^2+^ binding under 488-nm excitation, making it suitable for rapid dynamic imaging (Figure 2F) [40,43]. Although these dyes were initially developed for animal-cell studies, they were subsequently introduced into plant systems and provided an important methodological foundation for monitoring plant Ca^2+^ dynamics [4].

Chemical fluorescent dyes have several limitations in plant tissues. The cell wall restricts indicator loading, while light scattering and autofluorescence in intact tissues increase background signals. Some dyes also undergo compartmentalization, leakage, or active efflux after entering the cell, thereby compromising signal stability and quantitative reliability. Consequently, these indicators are most suitable for isolated cells, protoplasts, and local tissue preparations, whereas their use for whole-plant observation, long-term imaging, and high-resolution subcellular analysis is limited [2,4].

Unlike chemical fluorescent dyes, aequorin (AQ) is a bioluminescent Ca^2+^ indicator that does not require external excitation. Apoaequorin is reconstituted with the cofactor coelenterazine in the presence of oxygen; subsequent Ca^2+^ binding triggers coelenterazine oxidation and blue-light emission near 469 nm (Figure 2C). Expression of AQ in plants enabled whole-plant monitoring of Ca^2+^ dynamics and provided a favorable signal-to-noise ratio for continuous recording of global responses to cold, salinity, mechanical stimulation, and pathogen-related treatments [9]. Aequorin has also been widely used for stimulus-response analysis and mutant screening in plant Ca^2+^ research [2].

Aequorin also has clear limitations. Its luminescence is weak and its spatial resolution is limited, making it unsuitable for high-resolution subcellular imaging. Experiments generally require exogenous supplementation with the cofactor coelenterazine, which increases operational complexity. Accordingly, AQ is well suited for monitoring overall Ca^2+^ response trends but is less suitable for resolving local Ca^2+^ microdomains.

Together, chemical fluorescent dyes and aequorin established the early technical foundation of plant Ca^2+^ imaging. The subsequent development of genetically encoded Ca^2+^ indicators was driven largely by the limited tissue compatibility and spatial resolution of these earlier approaches [2,9].

### 3.2. Ca^2+^ Imaging with Ratiometric Fluorescent Indicators

The development of GECIs marked a new stage in plant Ca^2+^ imaging. Unlike chemical dyes, GECIs can be stably expressed through genetic transformation in specific tissues, cell types, or subcellular compartments, thereby avoiding the difficulties of exogenous indicator loading and improving experimental reproducibility. Among the earliest widely used GECIs were the Cameleon indicators, which are based on fluorescence resonance energy transfer (FRET). Cameleon indicators typically comprise cyan fluorescent protein (CFP) as the FRET donor, calmodulin (CaM) as the Ca^2+^-binding component, the M13 peptide as the CaM-interacting component, and Venus as the FRET acceptor. These components are arranged in a modular FRET-based architecture, with CaM and M13 positioned between CFP and Venus. Ca^2+^ binding induces a conformational change in the sensing module, bringing the donor and acceptor closer together and thereby altering FRET efficiency, which produces a quantitative change in the fluorescence ratio between the two channels (Figure 2A) [41].

Ratiometric indicators are quantified primarily using the fluorescence ratio between two channels rather than a single absolute fluorescence intensity. This reduces the influence of differences in expression level, local indicator concentration, minor focal drift, and partial photobleaching. Such robustness is particularly valuable in plant samples, which often continue to grow and move during imaging and commonly exhibit strong background fluorescence. Continued optimization of the Cameleon family has produced indicators with different Ca^2+^ affinities and subcellular targeting sequences for imaging the cytosol, nucleus, mitochondria, chloroplasts, and endoplasmic reticulum [44,45].

The effective imaging depth depends strongly on tissue optical properties and the microscopy platform. In *Arabidopsis* roots, conventional confocal imaging provides the clearest signals in relatively superficial cell layers, whereas signals from deeper cell types can be obscured by overlying fluorescence [46]. Two-photon excitation microscopy can improve visualization in deeper regions of intact plant tissues and reduce plant autofluorescence when suitable longer excitation wavelengths are used [47].

In plant research, ratiometric GECIs have greatly facilitated analysis of Ca^2+^ dynamics at the subcellular scale, particularly for comparisons among compartments. Subcellularly targeted Cameleon sensors have been used to characterize the temporal sequence of Ca^2+^ changes in the cytosol, nucleus, mitochondria, chloroplasts, and endoplasmic reticulum, providing direct evidence for compartmentalized Ca^2+^ signaling in plant cells [48].

Compared with bright single-fluorescent-protein indicators, FRET-based ratiometric indicators are generally dimmer, require more complex image acquisition and analysis, and place greater demands on instrumentation and experimental control. Their temporal resolution is also often lower than that of high-performance single-fluorescent-protein indicators, limiting their ability to resolve very rapid Ca^2+^ transients [49]. Overall, ratiometric GECIs are most advantageous when quantitative stability and resistance to imaging artifacts are priorities.

### 3.3. Ca^2+^ Imaging with Single-Fluorescent-Protein Indicators

Single-fluorescent-protein GECIs are characterized by high brightness and a broad dynamic range, with the GCaMP family being the best-known example. These indicators have an M13-cpEGFP-CaM architecture. When Ca^2+^ binds to the C-terminal CaM and promotes intramolecular interaction with the N-terminal M13 peptide, the three-dimensional barrel of the intervening circularly permuted enhanced green fluorescent protein (cpEGFP) is stabilized in a closed conformation. This markedly alters the local chromophore environment and enhances fluorescence (Figure 2B) [42]. Because only a single fluorescence channel is required, these indicators offer high temporal resolution and efficient data acquisition and processing.

Progressive optimization of the GCaMP family has improved sensitivity, brightness, response kinetics, and signal-to-noise ratio, enabling the recording of rapid Ca^2+^ transients [50,51]. In plants, these indicators have been widely applied to root tips, guard cells, pollen tubes, and wound-induced systemic Ca^2+^ signaling. Red fluorescent indicators such as R-GECO1 and jRGECO1a use circularly permuted red fluorescent proteins to generate Ca^2+^-dependent red signals (Figure 2D), expanding the options for multicolor imaging and combination with spectrally compatible green fluorescent reporters [52,53].

Most single-fluorescent-protein GECIs are non-ratiometric, and their fluorescence intensity is influenced by expression level, sample thickness, focal drift, photobleaching, and tissue movement. During long-term imaging or continuous tissue growth, quantification based solely on absolute fluorescence can therefore be biased, and motion artifacts can complicate signal interpretation. Although these indicators offer high sensitivity and temporal resolution, they should be used cautiously in rigorous quantitative analyses and cross-sample comparisons [50,51]. To address these limitations, ratiometric GECIs such as GCaMP-R and MatryoshCaMP6s have been developed to provide an internal fluorescence reference and improve quantitative reliability [54,55]. In plants, the CamelliA toolset enables the simultaneous imaging of Ca^2+^ dynamics in multiple subcellular compartments using spectrally distinct and compartment-targeted GECIs [56].

### 3.4. Principles for Selecting Ca^2+^ Indicators for Live Imaging

As the range of available Ca^2+^ indicators has expanded, the central question has shifted from whether a signal can be detected to whether indicator performance is appropriately matched to the biological problem. Indicator selection should consider Ca^2+^ affinity, dynamic range, response kinetics, and readout mode, together with the Ca^2+^ concentration range, pH, ionic environment, and autofluorescence of the target tissue or subcellular compartment [44,45]. Basal free Ca^2+^ levels differ markedly among compartments. The cytosol and nucleus generally require medium- to high-affinity indicators, whereas Ca^2+^-rich stores such as the vacuole and endoplasmic reticulum require lower-affinity sensors to avoid saturation. With appropriately targeted low-affinity indicators, decreases in luminal Ca^2+^ can also be resolved. For example, an endoplasmic-reticulum-targeted Yellow Cameleon with lower Ca^2+^ affinity detected a decrease in ER Ca^2+^ following inhibition of ER-type Ca^2+^-ATPases in *Arabidopsis* pollen tubes [57]. Intracellular pH changes, autofluorescence, and ionic composition can also alter indicator performance, so the same indicator may behave differently among tissues and compartments.

The optimal indicator also depends on the experimental objective. Bright single-fluorescent-protein indicators are often preferable for rapid transients or large-scale propagation events, whereas ratiometric indicators are advantageous when quantitative stability and reduced sensitivity to motion artifacts or changing imaging conditions are essential [48]. Thus, there is no universally optimal Ca^2+^ indicator; selection requires a balance among the requirements of the specific biological question.

Practical considerations such as experimental cost, operational complexity, and sensor stability should also be taken into account. Chemical fluorescent dyes are relatively straightforward to apply because they do not require generation of transgenic reporter lines, but repeated dye loading and intracellular leakage, compartmentalization, or active efflux can reduce signal stability [2,4]. Aequorin provides low-background genetically encoded reporting but requires coelenterazine supplementation and produces relatively weak luminescence [2,9]. In contrast, GECIs require genetic transformation and establishment of reporter lines, which increases the initial experimental effort; once established, however, they avoid repeated exogenous indicator loading and support reproducible long-term and cell-type- or compartment-specific imaging [45].

Subcellular targeting has further broadened the application of plant GECIs. By attaching appropriate targeting sequences, indicators can be directed to the nucleus, mitochondria, chloroplasts, endoplasmic reticulum, and other membrane-bound compartments, allowing comparative analysis of the temporal order and coupling of Ca^2+^ dynamics among compartments. Plant Ca^2+^ imaging has consequently progressed from asking whether a signal changes to determining where and when it changes and how different compartments coordinate their responses. The core photophysical parameters, strengths, limitations, and typical applications of commonly used plant Ca^2+^ indicators are summarized in Table 1.

### 3.5. Practical Considerations for Quantitative Ca^2+^ Imaging in Plants

Reliable quantitative Ca^2+^ imaging requires control of both sensor-related and imaging-related sources of variation. First, the affinity and dynamic range of the indicator should be matched to the expected Ca^2+^ concentration of the target compartment. Sensors optimized for cytosolic Ca^2+^ may become saturated in Ca^2+^-rich compartments, whereas low-affinity indicators may fail to resolve small cytosolic transients. Ratiometric indicators are generally preferable when quantitative comparisons among samples, developmental stages, or long imaging periods are required because an internal reference can partially compensate for differences in indicator abundance, focal drift, and sample movement [44,45,48,57].

Sample preparation should minimize mechanical perturbation, because mounting, compression, transfer between solutions, or changes in perfusion can themselves evoke Ca^2+^ signals. Experimental samples should therefore be allowed to equilibrate after mounting whenever possible, and untreated or mock-treated controls should be imaged under identical conditions. Particular care is required for growing tissues such as roots, pollen tubes, and guard cells, in which displacement and shape changes can alter fluorescence independently of Ca^2+^ concentration [9,45].

Imaging conditions should also be kept as constant as possible across experiments. Excitation intensity, detector gain, exposure time, optical section thickness, and acquisition frequency can all influence apparent signal amplitude. Excessive excitation should be avoided during long-term imaging because photobleaching and phototoxicity can progressively alter both fluorescence intensity and cellular physiology. When rapid Ca^2+^ transients are expected, acquisition frequency should be sufficiently high to resolve signal kinetics without introducing unnecessary light exposure [44,45].

Motion correction and consistent region-of-interest selection are particularly important for quantitative analysis. For non-ratiometric indicators, fluorescence changes are commonly expressed relative to a baseline value rather than as raw fluorescence intensity, whereas ratiometric sensors provide an internal normalization strategy that is less sensitive to variation in expression level and sample thickness. Background subtraction, baseline definition, normalization procedures, and criteria for identifying Ca^2+^ peaks should be applied consistently across all samples and reported explicitly [44,48].

**Figure 3:**
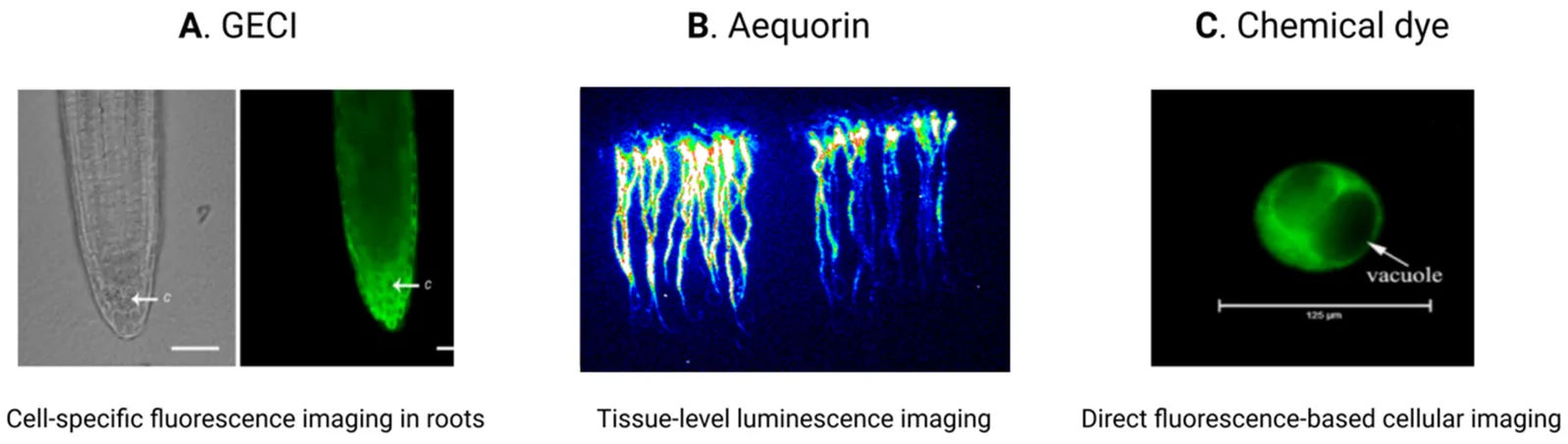
Representative visualization of plant Ca^2+^ signals using different classes of Ca^2+^ indicators. (**A**) Genetically encoded Ca^2+^ indicator (GECI)-based fluorescence imaging in *Arabidopsis thaliana* roots, illustrating cell-specific visualization of Ca^2+^ signals. (**B**) Aequorin-based pseudocolor luminescence imaging in *Arabidopsis thaliana* seedlings, illustrating tissue-level detection of Ca^2+^-dependent bioluminescence. (**C**) Fluo-8/AM-based chemical fluorescent dye imaging in *Malus domestica* protoplasts, illustrating direct fluorescence-based detection of cellular Ca^2+^ signals, Scale bars = 125 μm. Panel (**A**) was adapted from Krogman et al. [46] under the CC BY 4.0 license; panel (**B**) shows original unpublished imaging data generated by the authors; and panel (**C**) was adapted from Qiu et al. [59] under the CC BY 4.0 license. Panels (**A**,**C**) were cropped and incorporated into this composite figure. Panel (**B**) is included to illustrate aequorin-based bioluminescence imaging of Ca^2+^ signals at the tissue level in Arabidopsis seedlings, providing a representative example of whole-seedling Ca^2+^ visualization and complementing the fluorescence-based imaging approaches shown in panels (**A**,**C**).

Finally, direct comparison of absolute Ca^2+^ values among different indicators, tissues, or studies should be made cautiously unless appropriate calibration has been performed. Sensor affinity, pH sensitivity, temperature, ionic environment, expression level, and optical settings can all affect the measured response. Quantitative interpretation is therefore most reliable when sensor selection, sample preparation, imaging parameters, calibration, motion correction, and data-processing procedures are standardized within the same experimental framework [44,45,48,57]. Representative examples of plant Ca^2+^ imaging using genetically encoded indicators, aequorin, and chemical fluorescent dyes are shown in Figure 3.

## 4. Advances in the Applications of Plant Ca^2+^ Imaging

Improved GECIs and imaging platforms now allow plant Ca^2+^ signals to be resolved across subcellular, cellular, and tissue scales. These tools have shifted the field from detecting whether Ca^2+^ changes occur to comparing when, where, and how signals develop under different physiological conditions. Applications now span abiotic stress, immunity, development, and systemic signaling [3,14]. Live Ca^2+^ imaging has been applied to a wide range of plant species, including *Arabidopsis thaliana*, *Oryza sativa*, *Solanum lycopersicum*, *Solanum tuberosum*, and *Medicago truncatula*, to investigate abiotic stress perception, plant immunity, growth and development, and long-distance systemic signaling. Representative studies and applications are summarized in Table 2.

### 4.1. Imaging Ca^2+^ Dynamics During Abiotic Stress

Exposure to low temperature, salinity, drought, heat, or mechanical stimulation often elicits rapid Ca^2+^ responses within seconds to minutes. Early aequorin-based studies showed that cold and mechanical stimulation rapidly increase cytosolic Ca^2+^ in *Arabidopsis* and that the waveform differs among stimuli, providing experimental support for the concept of Ca^2+^ signatures [9,10]. With continued advances in live imaging, the spatial distribution and intertissue propagation of Ca^2+^ signals during abiotic stress have become increasingly clear.

During salt stress, local application of salt to roots can induce Ca^2+^ waves that propagate through tissues and spread systemically to the shoot, thereby coordinating whole-plant stress responses [71]. Ca^2+^ imaging has also revealed strong cell-type specificity in stress perception and response. Different regions of the root apex, guard cells, and epidermal cells display distinct Ca^2+^ dynamics during osmotic perturbation, indicating that abiotic stress-associated Ca^2+^ signals are jointly shaped by cellular specialization and the local tissue environment [3,14]. Combining forward genetics with high-resolution Ca^2+^ imaging has identified specific sensors of mechanical and osmotic stress. For example, characterization of the OSCA family, including *Arabidopsis* OSCA1 and its rice homolog OsOSCA1.1, and cyclic nucleotide-gated channels such as OsCNGC14 and OsCNGC16, has provided important molecular evidence for mechanosensing, thermal responses, and osmotic regulation in plants (Table 2).

### 4.2. Visualization of Ca^2+^ Signals in Plant Immunity

Ca^2+^ signaling is one of the earliest events in plant immunity. When microbe-associated molecular patterns (MAMPs) are perceived by pattern-recognition receptors, Ca^2+^ rapidly enters the cytosol across the plasma membrane and initiates downstream responses, including the oxidative burst, mitogen-activated protein kinase (MAPK) cascades, and defense-gene expression [11,12]. High-performance GECIs now allow these dynamics to be observed directly in living leaves and roots.

Different MAMPs generate distinct Ca^2+^ responses with characteristic peak amplitudes, durations, and recovery kinetics. At the tissue scale, MAMP- and DAMP-induced Ca^2+^ waves initiate in subsets of epidermal cells and propagate slowly at an approximately constant rate, whereas wound-induced waves spread more rapidly but decelerate with increasing distance, indicating that distinct stimuli engage different mechanisms of Ca^2+^ wave propagation [72]. Receptor mutations or loss of relevant ion channels often weaken, delay, or abolish these signals [11]. Ca^2+^ imaging can therefore reveal whether an immune response has been activated and help clarify how receptor recognition is coupled to ion-channel regulation and downstream signaling.

Live Ca^2+^ imaging has also provided direct evidence linking local and systemic immunity. Mechanistically, the Ca^2+^-responsive CBL1–CIPK26/CPK5 bi-kinase module sensitizes and synergistically activates the NADPH oxidase RBOHD, enabling relatively small Ca^2+^ elevations to reinforce coupled Ca^2+^–ROS signal propagation and systemic immune responses [73]. Following local biotic or physical stimulation, Ca^2+^ increases may occur not only at the stimulated site but also in neighboring and distal tissues, indicating that plant defense involves coordinated signaling across multiple tissues rather than responses confined to a single site [12,17]. This approach therefore provides an important means of understanding how local recognition develops into whole-plant defense. Live imaging has also contributed to the analysis of disease resistance in crops. In rice, for example, Ca^2+^ influx mediated by cyclic nucleotide-gated channels such as OsCNGC9 is a central step in activating downstream immune outputs, including the oxidative burst and defense-gene expression. These imaging-based studies have greatly refined our understanding of plant immune signaling networks (Table 2).

### 4.3. Ca^2+^ Dynamics During Development and Polar Growth

Ca^2+^ dynamics are important not only in stress responses but also during normal plant growth and development. Polar growth is among the earliest and most extensively studied applications of live Ca^2+^ imaging. Distinct Ca^2+^ gradients and oscillations occur at the tips of pollen tubes and root hairs, where they are closely associated with vesicle trafficking, cell-wall remodeling, and directional elongation [74]. Ca^2+^ imaging therefore provides direct evidence for the ionic dynamics and regulatory mechanisms that operate in regions of polar growth.

During stomatal movement, Ca^2+^ oscillations in guard cells are important components of abscisic acid (ABA) signaling. ABA-induced Ca^2+^ dynamics regulate membrane ion channels, alter guard-cell turgor, and thereby control stomatal opening and closure, with consequences for transpiration and gas exchange [13,75]. Ca^2+^ signaling thus provides an important link between developmental regulation and environmental response.

The use of subcellularly targeted indicators has extended the study of development-related Ca^2+^ signals to specific organelles. Distinct Ca^2+^ dynamics in the nucleus, chloroplasts, and mitochondria are associated with cell-cycle regulation, metabolic state, and coordination of cellular functions [48]. These findings indicate that Ca^2+^ regulation during plant development is compartmentalized and that signals in different subcellular spaces may encode distinct physiological functions.

### 4.4. Long-Distance Systemic Ca^2+^ Signaling

The direct visualization of long-distance systemic Ca^2+^ propagation represents a major advance in plant Ca^2+^ imaging. Although plants lack a nervous system, vascular-associated tissues support rapid long-distance communication. Live imaging has shown that local physical damage, including herbivore feeding, and localized salt stress can rapidly induce Ca^2+^ waves that propagate through vascular-associated tissues and transmit information to distal organs [17,71]. These observations provide direct evidence for rapid long-distance signaling in plants. Ca^2+^ is particularly well suited to this role because its resting cytosolic concentration is maintained at a low level relative to extracellular and intracellular Ca^2+^ stores, creating steep gradients that permit rapid, reversible, and spatially restricted changes after channel activation [1,2,3,4]. This signaling architecture helps explain why Ca^2+^, rather than other divalent ions, is widely used as a dynamic second messenger in these pathways.

Long-distance Ca^2+^ waves are commonly coupled to membrane depolarization, reactive oxygen species (ROS) waves, and hormone signaling, particularly jasmonate-related pathways. Wound-released glutamate activates GLUTAMATE RECEPTOR-LIKE channels that contribute to both leaf-to-leaf electrical signaling and Ca^2+^ influx, indicating that the systemic response is not a Ca^2+^-only process [17,76]. Changes in K^+^ and other cation fluxes can also contribute to membrane-potential dynamics associated with plant electrical signaling [18,67]. In distal tissues, these coupled signals activate Ca^2+^ decoders and induce downstream transcriptional reprogramming, thereby establishing systemic defense responses throughout the plant [14,17]. Ca^2+^ imaging is particularly valuable because it reveals not only whether signal propagation occurs but also its temporal and spatial dynamics, providing direct evidence for the kinetics of rapid whole-plant communication.

In addition to vascular-associated pathways, plasmodesmata provide a symplastic route for cell-to-cell communication. Each plasmodesma contains a narrow cytoplasmic sleeve surrounding an endoplasmic-reticulum-derived desmotubule, and its permeability is dynamically regulated by callose deposition at the neck region. Recent work showed that Ca^2+^ signals and CALMODULIN-LIKE 41 participate in dsRNA-induced callose deposition at plasmodesmata, directly linking Ca^2+^ signaling to the regulation of symplastic connectivity [77]. Thus, plasmodesmatal gating may modulate the cell-to-cell spread of signaling molecules and complement vascular, electrical, and Ca^2+^-wave pathways during local and systemic stress responses.

## 5. Conclusions and Future Perspectives

Plant Ca^2+^ signaling is generated and shaped by the coordinated activities of plasma-membrane channels, intracellular Ca^2+^ stores, pumps, exchangers, and organelle-associated transport systems. Advances from chemical fluorescent dyes and aequorin to ratiometric and single-fluorescent-protein genetically encoded calcium indicators have enabled Ca^2+^ dynamics to be visualized across subcellular, cellular, tissue, and whole-plant scales. These approaches have substantially improved our understanding of Ca^2+^ signaling in abiotic stress responses, plant immunity, polar growth, development, symbiotic interactions, and long-distance systemic communication.

Despite these advances, several technical and conceptual limitations remain. Indicator affinity, dynamic range, kinetics, and spectral properties must be appropriately matched to the Ca^2+^ concentration, pH, and ionic environment of the target tissue or subcellular compartment. Future progress will require not only improved sensor performance but also standardized imaging and analytical frameworks. Such advances will enhance the quantitative interpretation and comparability of plant Ca^2+^ imaging studies, while broader spectral palettes, improved targeting strategies, and computational approaches will facilitate the dissection of how specific transport systems generate and decode Ca^2+^ signatures.

## Figures and Tables

**Table 1:** Core photophysical properties and representative applications of commonly used Ca^2+^ indicators in plants.

Indicator	Category	*K* *_d_*	Key Features	Representative Applications	Representative References
Fura-2	Chemical dye (ratiometric)	224 nM	Dual-excitation ratiometric readout with strong quantitative performance; suitable for cellular Ca^2+^ measurements.	Quantitative analysis of Ca^2+^ in single cells or isolated cell systems.	[40]
Fluo-4	Chemical dye (non-ratiometric)	345 nM	Large fluorescence response and relatively simple acquisition; suitable for rapid dynamic imaging.	Monitoring rapid cellular Ca^2+^ dynamics	[43]
Aequorin	Bioluminescent protein	Variable	Low background and suitable for tissue- and whole-plant-scale Ca^2+^ measurements.	Monitoring stress-induced Ca^2+^ signals in seedlings and intact tissues.	[9]
YC3.6 (Yellow Cameleon 3.6)	FRET-based indicator (ratiometric)	~250 nM	Ratiometric readout reduces the effects of sample displacement and differences in indicator expression.	Long-term in situ imaging during root development and plant–microbe interactions.	[48,58]
GCaMP family	Single fluorescent protein (green)	Variant-specific	Bright signal, rapid response, and convenient targeting to specific cell types and subcellular compartments.	Live imaging of stimulus-induced Ca^2+^ dynamics.	[40,48,57]
R-GECO1	Single fluorescent protein (red)	480 nM	Compatible with multichannel imaging and can be combined with green fluorescent reporters.	Simultaneous imaging of Ca^2+^ and other signaling molecules.	[52]

*K_d_*, dissociation constant, indicates the apparent affinity of an indicator for Ca^2+^; a lower *K_d_* generally corresponds to a higher Ca^2+^ affinity.

**Table 2:** Applications of live Ca^2+^ imaging in plant environmental responses, growth, and development.

Research Context	Species	Detection Method/Indicator	Spatial Scale Resolved	Main Findings	Representative Reference
Heat stress	*Oryza sativa*	NES-YC3.6	Cellular level	High temperature induces characteristic Ca^2+^ signals; OsCNGC14 and OsCNGC16 contribute to heat-tolerance responses.	[60]
Root salt-stress sensing	*Oryza sativa*	YC3.6	Cellular level	OsOSCA1.1 participates in root salt-stress sensing and mediates increases in cytosolic Ca^2+^.	[61]
ABA-induced stomatal closure	*Arabidopsis thaliana*	R-GECO1-mTurquoise	Cellular level	Guard-cell Ca^2+^ transients occur predominantly during stomatal closure and contribute to ABA-induced stomatal regulation.	[62]
Cold and freezing stress	*Arabidopsis thaliana*	Aequorin	Tissue level	A rapid decrease in temperature triggers a sharp cytosolic Ca^2+^ increase; ANNEXIN1 contributes to cold-induced Ca^2+^ signaling.	[63]
High-light stress	*Arabidopsis thaliana*	Fluo-4-AM	Tissue/whole-plant level	Local high light triggers systemic Ca^2+^ waves; HPCA1 contributes to this response and to stress acclimation.	[64]
Herbivore defense	*Arabidopsis thaliana*	GCaMP3	Tissue/whole-plant level	Herbivore feeding induces GLR-dependent systemic Ca^2+^ waves that contribute to long-distance defense signaling.	[17]
Pollen-tube polar growth	*Solanum lycopersicum*	YC3.6	Cellular/subcellular level	A dynamic Ca^2+^ gradient is maintained at the pollen-tube tip and is associated with polar growth.	[65]
Nodule symbiosis signaling	*Medicago truncatula*	Nuclear-targeted Cameleon (NupYC2.1)	Subcellular (nucleus)	Nod factors induce rhythmic nuclear Ca^2+^ oscillations, supporting a central role for nuclear Ca^2+^ signals in symbiotic regulation.	[66]
Heat-shock-induced systemic signaling	*Solanum tuberosum*	Aequorin	Tissue level	Heat-shock-induced electrical signals are accompanied by cytosolic Ca^2+^ transients and are associated with systemic defense responses.	[67]
Osmotic stress sensing	*Arabidopsis thaliana*	Aequorin	Tissue level	OSCA1 contributes predominantly to osmotic-stress-induced cytosolic Ca^2+^ increases and helps distinguish osmotic from ionic stress perception.	[68]
H_2_O_2_ sensing/oxidative stress	*Arabidopsis thaliana*	Aequorin/YC3.6	Tissue and cellular level	HPCA1, a plasma-membrane LRR receptor kinase, senses extracellular H_2_O_2_ and triggers the Ca^2+^ influx required for ROS signaling.	[69]
Salt-ion sensing	*Arabidopsis thaliana*	Aequorin/YC3.6	Tissue and cellular level	MOCA1-dependent GIPC sphingolipids participate in Na^+^ sensing and are required for salt-induced membrane depolarization, Ca^2+^ spikes and waves, SOS-pathway activation, and growth regulation.	[70]

## Data Availability

No new data were created or analyzed in this study. Data sharing is not applicable to this article.
